# Green manuring combined with nitrogen reduction sustained rice productivity and expanded soil organic nitrogen pool via promoting coordinated rhizosphere metabolite–microbiome interactions

**DOI:** 10.3389/fpls.2026.1966893

**Published:** 2026-09-30

**Authors:** Hai Liang, Zhongyi Li, Caihui Wei, Hongqin Tang, Wenbin Dong, Yuefeng Yu, Yuning Wang, Yongcheng Mo, Songjuan Gao, Weidong Cao, Tieguang He

**Affiliations:** 1 Agricultural Resources and Environmental Research Institute, Guangxi Academy of Agricultural Sciences/Guangxi Key Laboratory of Arable Land Conservation, Nanning, Guangxi, China; 2 College of Resources and Environmental Sciences, Nanjing Agricultural University, Nanjing, China; 3 State Key Laboratory of Efficient Utilization of Arid and Semi-arid Arable Land in Northern China, the Institute of Agricultural Resources and Regional Planning, Chinese Academy of Agricultural Sciences, Beijing, China

**Keywords:** green manure, metabolites, microbiome, nitrogen reduction, soil organic nitrogen

## Abstract

Green manuring can improve soil fertility and sustain rice productivity, yet its effects on rhizosphere metabolite–microbiome interactions regulating soil nitrogen pools remain unclear. Based on a six-year field experiment in the green manure-rice rotation system, we investigated how green manure combined with reduced nitrogen fertilization regulated soil nitrogen pool, enzyme activities, rhizosphere metabolites and microbial communities along the soil–root continuum. Green manuring combined with 20% reduction of nitrogen fertilizer (GM2) increased rice yield by approximately 8.7% and effectively expanded soil organic nitrogen pool. In contrast, green manuring without nitrogen reduction (GM3) enhanced inorganic nitrogen accumulation but accelerated organic nitrogen depletion. GM2 promoted the accumulation of soil amino sugar nitrogen, amino acid nitrogen and the activities of organic nitrogen-hydrolyzing enzymes (protease, leucine aminopeptidase and β-N-acetylglucosaminidase), indicating accelerated organic nitrogen depolymerization and turnover. Green manuring (GM) exerts an increasing influence on microbial community assembly along the soil–root continuum and promotes nitrogen-cycling functional potentials, most notably in the root endosphere of GM3. GM treatments enriched amino acids and signaling-related metabolites of rice rhizosphere soil, including L-isoleucine, sarcosine, 3-[(2-chloro-1,3-thiazol-5-yl) methyl]-5-methyl-1,3,5-oxadiazin-4-imine, and N-sulfo-D-glucosamine, all of which showed significant positive associations with nitrogen-cycling related bacterial taxa and saprotrophic fungi. Path modeling further demonstrated that metabolite-driven bacterial pathways dominated soil organic nitrogen accumulation. Overall, moderate nitrogen reduction under green manuring promoted coordinated rhizosphere metabolite–microbiome interactions that simultaneously sustained rice productivity and improved soil nitrogen stability.

## Introduction

The application of nitrogen (N) fertilizers in agricultural systems around the world has increased dramatically, from 11.5 million tons in 1961 to 111.6 million tons in 2023 with N fertilization showing the largest increase (FAO, 2025). China is the world’s largest consumer of N fertilizer, with total national N fertilizer input increasing from 9.3 million tons in 1980 to 15.6 million tons in 2024 (National Bureau of Statistics of China, 2025). With the increase in fertilization, rice production of China has increased by approximately 52% over the past four decades (National Bureau of Statistics of China, 2025). However, rice typically recover less than 40% of applied N fertilizer, with more than half of it being lost to the surrounding environment (Xia et al., 2017; Xia and Yan, 2023).

The application of green manures in paddy fields in southern China severe as a way to optimal use the fallow fields, for which is used primarily as a sustainable agricultural regime. Excessive nitrogen application leads to farmland degradation, while green manure can replace some chemical fertilizers and improve soil quality (Gao et al., 2023). Incorporating leguminous green manure into the soil, combined with appropriate nitrogen reduction, can increase soil organic matter, microbial biomass, and nitrogen supply capacity (Yang et al., 2026). It also promotes the expression of genes involved in carbon and nitrogen cycling by regulating the microbial community structure (Liang et al., 2024). Plantation of milk vetch and hairy vetch after the rice harvest can produce nitrogen-rich organic biomass through their growth attributes, thereby providing essential nutrients for subsequent rice cultivation (Gao et al., 2021). Former studies have demonstrated that the leguminous green manures can increase subsequent crop yields by about 29% and contribute to the N fertilization decrease by 26% (Cernay et al., 2018; Gao et al., 2023). Green manure-rice rotation can maintain rice yield with a reduction of 20%-40% N fertilizer inputs (Liang et al., 2022; Xie et al., 2016), and beneficial for soil quality, enhances crop nitrogen uptake, and reduces reactive nitrogen losses (Zhang et al., 2023).

The primary contribution of leguminous green manures to the nitrogen supply in paddy fields lies in their biological N_2_ fixation and the subsequent return of residues. Crop uptake and utilization of green manure-derived nitrogen depend on the microbial degradation of macromolecular organic nitrogen compounds in the residues, including proteins and chitin. These compounds can stimulate the microbial turnover of the organic nitrogen pool, thereby potentially enhancing nitrogen availability. Studies have demonstrated that the transformations between different components of the soil organic nitrogen and inorganic nitrogen determine the efficiency of crop uptake (Chen et al., 2024). For instance, hydrolyzable organic nitrogen represents an active fraction, including low molecular weight organic nitrogen, such as amino acids and peptides, that can be directly absorbed by crops, while also serving as a vital source of soil ammonium (Liu et al., 2026).

In response to fluctuations in soil nutrient dynamics, plants do not passively endure unfavorable conditions, they proactively recruit specific functional microbial consortia through shifts in their root metabolic profiles, thereby bolstering their fitness (Fan et al., 2025; Sasse et al., 2018). The microbes recruited by plants inhabit various ecological niches, including both aboveground and belowground compartments, establishing a micro-habitat where their interaction with the host benefits the entire community. Belowground niches are typically categorized into bulk soil, rhizosphere, rhizoplane and endosphere depends on their proximity to the plant roots. The continuum of bulk soil-plant root plays a pivotal role in transporting and releasing photosynthates, providing a nutrient-rich habitat that supports the flourishing of surrounding microbes (Acharya et al., 2023). Typically, in response to plant cues, these microbes typically secrete a diverse array of extracellular enzymes, such as protease and urease, to break down complex organic nitrogen polymers in the soil, thereby facilitating a reciprocal resource exchange with the host plant. However, a critical knowledge gap remains in our understanding of how rice plants communicate with soil microbial communities to regulate nitrogen acquisition from soil organic matter. More importantly, it is still unclear whether and how rice plants signal their specific nitrogen demands to stimulate microbial degradation of complex organic nitrogen and thereby obtain bioavailable nitrogen forms required for growth. In this study, a 6-year field experiment (2019-2024) in southern China that focused on the integrated green manuring and nitrogen reduction optimal rice yields and soil healthy. This study focuses on two overarching objectives: (1) to characterize the changes in organic and inorganic nitrogen components in soil under green manuring with reduced nitrogen fertilization, and (2) to elucidate how rice plants regulate rhizosphere metabolic profiles to selectively recruit specific functional microbial communities, thereby responding to shifts in soil nitrogen pools and securing the nutrients required for growth.

## Methods

### Field experiment description

The long-term field experiment was initiated in June 2018 at the rice research station of the Guangxi Academy of Agricultural Sciences, located in Natong Town, Long’an County, Nanning, Guangxi, China (107°51′21″ E, 23°00′41″ N). The site is characterized by a subtropical monsoon climate, with a mean annual temperature of 21.6 °C, an annual precipitation of approximately 1300 mm, and an average elevation of 64 m. The soil at the experimental site had the following properties: pH 6.8, soil organic matter (SOM) 31.3 g kg^-^¹, total nitrogen (TN) 1.90 g kg^-^¹, alkali-hydrolyzable nitrogen 136.0 mg kg^-^¹, available phosphorus (AP) 19.4 mg kg^-^¹, and available potassium (AK) 161.0 mg kg^-^¹. The cropping system at the site consisted of a green manure–early rice–late rice rotation.

This experiment contains four treatments: winter fallow with conventional fertilization (F); Combination of green manure (milk vetch, *Astragalus sinicus* L.) and nitrogen reduction, with 40% nitrogen reduction (GM1), (3) 20% nitrogen reduction (GM2) and without nitrogen reduction (GM3). A randomized complete split-plot design was employed in this field, with each plot having an area of ​​20.7 m^2^. In the green manuring (GM) treatments, milk vetch was sown 7 days before the late rice harvest at a seeding rate of 30 kg ha^-1^. In mid-March of the following spring, milk vetch was incorporated into the paddy soil. The incorporation rate of Chinese milk vetch was consistent across the three GM treatments, at approximately 7500 kg ha^-^¹ of fresh biomass (equivalent to about 2250 kg ha^-^¹ of dry matter) at the full-flowering stage, with an average nitrogen content of 2.8%. After a persistent decomposition of milk vetch for approximately 3 weeks, early rice was transplanted in early April, and harvested in mid-July. About 20 days later, late rice was transplanted in early August and harvested in late October. The conventional fertilization regime for both early and late rice consisted of 180.0 kg ha^-1^ N (Urea, 46% N), 90.0 kg ha^-1^ P_2_O_5_ (Calcium superphosphate, 18.0% P_2_O_5_), and 120.0 kg ha^-1^ K_2_O (KCl, 60% K_2_O). And the nitrogen reduction management for each GM treatment was calculated based on the conventional fertilization regime. Phosphorus and potassium fertilizers were applied as basal doses, whereas nitrogen fertilizer was split into three applications, with 40%, 30%, and 30% allocated to basal, tillering, and panicle fertilizer, respectively.

### Soil sampling and measurement

A total of 40 soil samples (bulk soil and rhizosphere soil) and 20 plant root samples were collected On July 11, 2024 at the early rice harvest stage. Bulk soil samples were collected by excavating soil cores to a depth of 20 cm between rice rows using the manual soil sampler. Each individual bulk soil sample was divided into three parts: one part was used for TN, organic nitrogen components, total carbon (TC), pH value, AK, and AP quantified; another part was stored at -20 °C to determine activity indicators (inorganic nitrogen); and the third part was stored at -80 °C for organic nitrogen hydrolases measurement and DNA extraction and sequencing.

Rice plants with uniform growth were selected from each experimental plot and carefully excavated from the soil. Using sterile forceps and scissors, two to three tillers of the root were carefully excised from the cross-sections. These roots were then vigorously shaken in 30 mL of sterile deionized water until the soil particles attached to the root surface were detached. Subsequently, the roots were removed using sterile forceps and discarded. The remaining suspension was centrifuged at 3000 rpm for 15 min. The supernatant was partially removed until approximately 5 mL of the soil pellet and suspension remained, which were then thoroughly mixed. This mixture was designated as the rhizosphere soil samples and subsequently stored at -80 °C for DNA extraction, sequencing and metabolomics analysis.

To obtain high-quality root endophytic microbial data, the bulk soil attached to root was manually removed using sterile gloves, and three to four whole tillers of roots were retained. The roots were then rinsed with sterile deionized water until no visible soil particles remained. The cleaned roots were then immersed in 70% ethanol for surface sterilization, followed by repeated rinsing with sterile water to remove residual ethanol. The sterilized root segments were subsequently used for DNA extraction. This procedure was intended to remove microorganisms associated with the root surface and minimize the contribution of rhizoplane microorganisms to the endosphere samples. This washing process was repeated three times using fresh tubes. Residual moisture was removed from the roots using sterile filter paper. Finally, the roots were fragmented into 2 mm segments using sterile scissors; these segments were designated as the root endosphere samples.

Soil NO_3_^−^-N and NH_4_^+^-N (MIN) were extracted with 1 M KCl and analyzed using a continuous-flow analyzer (SAN++, Skalar, Netherlands). Basic soil chemical properties, including soil organic carbon (SOC) and total nitrogen (TN), were determined following the standardized protocols of Bao (2000). The components of soil organic nitrogen (SON), including Acid-hydrolyzable nitrogen (AHN), Ammonium nitrogen (AN), amino acid nitrogen (AAN), amino sugar nitrogen (ASN), non-hydrolyzable nitrogen (NHN) and hydrolyzable unidentified nitrogen (HUN), were determined by acid hydrolysis following the methods of Bremner (1965) and Stevenson (1982); see **Supplementary Materials** for detailed method.

Soil enzyme activities related to organic nitrogen cycling, including urease, protease, leucine aminopeptidase (LAP), and β-N-acetylglucosidase (NAG), were quantified using the microplate fluorescence-labeled substrate method (Ladd and Butler, 1972; Marx et al., 2001; Tabatabai and Bremner, 1972). Additional details on enzyme activity assays are provided in the **Supplementary Materials**.

### Amplicon sequencing and bioinformatic analysis

Total DNA was extracted from the soil and root samples using the FastDNA SPIN Kit for Soil (MP Biomedicals, CA, USA) according to the manufacturer’s instructions. DNA quality and concentration were verified via 1.0% agarose gel electrophoresis and spectrophotometry (Nanodrop ND-2000; Thermo Fisher, Waltham, MA, USA), respectively. Bacterial 16S rRNA gene libraries were generated using the primers 799F (5′-AACMGGATTAGATACCCKG-3′) and 1193R (5′-ACGTCATCCCCACCTTCC-3′) (Bai et al., 2015), while fungal ITS libraries were amplified using the primers ITS1 (5′-CTTGGTCATTTAGAGGAAGTAA-3′) and ITS2 (5′-GCTGCGTTCTTCATCGATGC-3′) (Blaalid et al., 2013). Amplicons were sequenced on the Illumina MiSeq PE300 platform (Allwegene, Beijing, China) using a paired-end protocol.

Raw sequences were processed using QIIME2, with the DADA2 plugin applied for quality control, denoising, chimera removal, and the generation of amplicon sequence variants (ASVs) (Callahan et al., 2016). Taxonomic assignment was performed using the SINA aligner against the SILVA database (v.138) for bacterial sequences (Pruesse et al., 2012) and the RDP classifier against the UNITE database (v.8.0) for fungal sequences. Functional annotation of prokaryotic taxa (FAPROTAX) was used to annotate the functional predictions of organic nitrogen cycling processes and FUNGuild was used to predict the saprophytic function of fungi. In total, 3603389 bacterial and 5922188 fungal high-quality sequences were obtained, yielding 52,365 and 9,159 ASVs, respectively, across all bulk soil, rhizosphere soil and root endosphere samples.

### Soil metabolite extraction, UHPLC-MS/MS and metabolite data analysis

Individual rhizosphere soil samples (100 mg) were carefully pretreated as follows: samples were homogenized using liquid N_2_, transferred to pre-chilled tubes containing 1 mL of extraction solvent (acetonitrile-methanol-water, 2:2:1, v/v/v), and vortexed for 30 s to ensure thorough suspension. The mixture was then homogenized with a ball mill at 45 Hz for 4 min and subjected to ultrasonic treatment on ice for 5 min. Following incubation at -20 °C for 1 h to precipitate proteins, the samples were centrifuged at 15000×g for 15 min at 4 °C. The resultant supernatants were carefully transferred to fresh tubes and evaporated to dryness under a vacuum. For UHPLC-MS/MS analysis, the dried extracts were reconstituted in 100 μL of a water-acetonitrile solution (1:1, v/v), vortexed thoroughly, and centrifuged again at 15000×g for 15 min at 4 °C. An equal volume of the final supernatants from all samples was pooled to serve as quality control (QC) samples, and the remaining supernatants were used for analysis. Metabolomic profiling was performed using an ultra-high performance liquid chromatography-tandem mass spectrometry (UHPLC-MS/MS) system, comprising a Vanquish UHPLC (Thermo Fisher, Germany) coupled with an Orbitrap QE HF-X mass spectrometer (Cheng et al., 2022).

Raw data were processed using Compound Discoverer 3.1 (Thermo Fisher) for peak alignment, retention time correction, and peak area extraction. Metabolite identification was achieved by matching exact masses (<10 ppm) and secondary fragmentation patterns against databases, including the Kyoto Encyclopedia of Genes and Genomes (KEGG) and the Human Metabolome Database (HMDB). Comprehensive metabolite annotation and subsequent statistical analyses followed the procedures described by Cheng et al. (2022).

A total of 12888 metabolites were identified from the rice rhizosphere, of which 1722 were clearly annotated (**Supplementary Table S1**; **Supplementary Figures S20–23**). These metabolites were classified into four levels (superclass, class, subclass and individual metabolite), with a total of 197 subclasses summarized (**Supplementary Table S2**). Metabolic pathway characterization using the Kyoto Encyclopedia of Genes and Genomes (KEGG) database revealed that the predominantly enriched pathways were amino acid metabolism, the biosynthesis of secondary metabolites, and biodegradation (**Supplementary Figure S15**). The subsequent random forest analysis, co-occurrence network analysis and partial least squares path modeling (PLS-PM) were exclusively performed based on the annotated metabolites to ensure results interpretability.

Multivariate statistics were conducted in SIMCA (v17.0.1) to generate partial least squares discriminate analysis (PLS-DA) models. PLS-DA was performed using annotated and unannotated metabolites using Pareto-scaled data. The list of scores and loading coordinates were then plotted in GraphPad Prism. Prism was used to visualize PLS-DA scores and PLS-DA loadings.

### Statistical analysis

One-way analysis of variance (ANOVAs) conducted to determine the dependence of crop yield and nutrient content, soil physicochemical properties and soil enzyme activity on different treatments. The constrained analysis of principal coordinates (CAP) testing the effect of the different treatments on the microbiome across rice soil-root continuum. All ordination analyses were performed using the R package phyloseq. Statistical significance of the CAP was assessed using the permutest function in the vegan package with 10^4^ permutations. We tested for agricultural practice effects on microbial community dissimilarity with permutational analysis of variance (PERMANOVA) and permutational analysis of multivariate dispersions (BETADISP) using the functions adonis and betadisp, respectively, in the “vegan” package with 10^4^ permutations (Oksanen et al., 2019). Where applicable, pairwise differences between the agricultural practice were assessed with the function pairwise perm manova from the package “RVAideMemoire”.

Random forest analysis was performed using the “randomForest” package (Liaw and Wiener, 2002) to identify the most important metabolites associated with soil organic and inorganic nitrogen accumulation. The response variable was subjected to 500 permutations using the “A3” package to assess the significance of the model. The significance of the contribution of metabolism to the soil nitrogen pool was assessed using the “rfPermute” package (Delgado-Baquerizo et al., 2016).

The relationships between rhizosphere metabolite composition, microbial community composition, and functional microbial population were revealed by the Procrustes analysis coupling with Mantel test. Regression analysis based on the Bray-Curtis dissimilarity was carried out to investigate the relationship of rhizosphere metabolite composition to microbial community composition and functional microbial population.

R and Gephi tools were used to create the ecological network in order to decode the multilevel relationships between rhizosphere metabolite components, metabolites and nitrogen cycling related bacteria, metabolites and saprophytic fungi members. Using the “psych” package, all potential Spearman’s rank correlation coefficients were generated to analyze the interacting networks. Data on the correlation was filtered with a fixed-point of 0.85 for absolute r. To increase the network’s authenticity, the data from the previous stage and edges with merged *P* < 0.05 were kept after applying Benjamini-Hochberg’s false discovery rate modification (Benjamini and Hochberg, 1995).

Then mantel tests were further performed using the package “vegan” to determine the correlation between potential metabolites and microorganisms associated with the accumulation of organic and inorganic nitrogen with soil properties and crop nutrients. PLS-PM was conducted using the “plspm” package in R. The model was performed with the presumed relationships to disentangle the direct and indirect effects of the metabolites and microbes on soil AHN and MIN accumulation.

## Results

### Rice yield and nutrients uptake

The six-year average yield showed that GM2 and GM3 significantly improved, while GM1 maintained grain yield comparable to that in F (**Figure 1**). Specifically, no significant differences in rice yield were observed between the three GM treatments and the F treatment during 2019 and 2020. From 2021 to 2023, GM3 significantly outyielded F, whereas GM1 and GM2 maintained stable production. By 2024, GM2 and GM3 increased grain yield by 8.7% and 11.2% relative to F (6728 kg ha^-1^), respectively, while GM1 continued to exhibit a stable yield performance.

**Figure 1:**
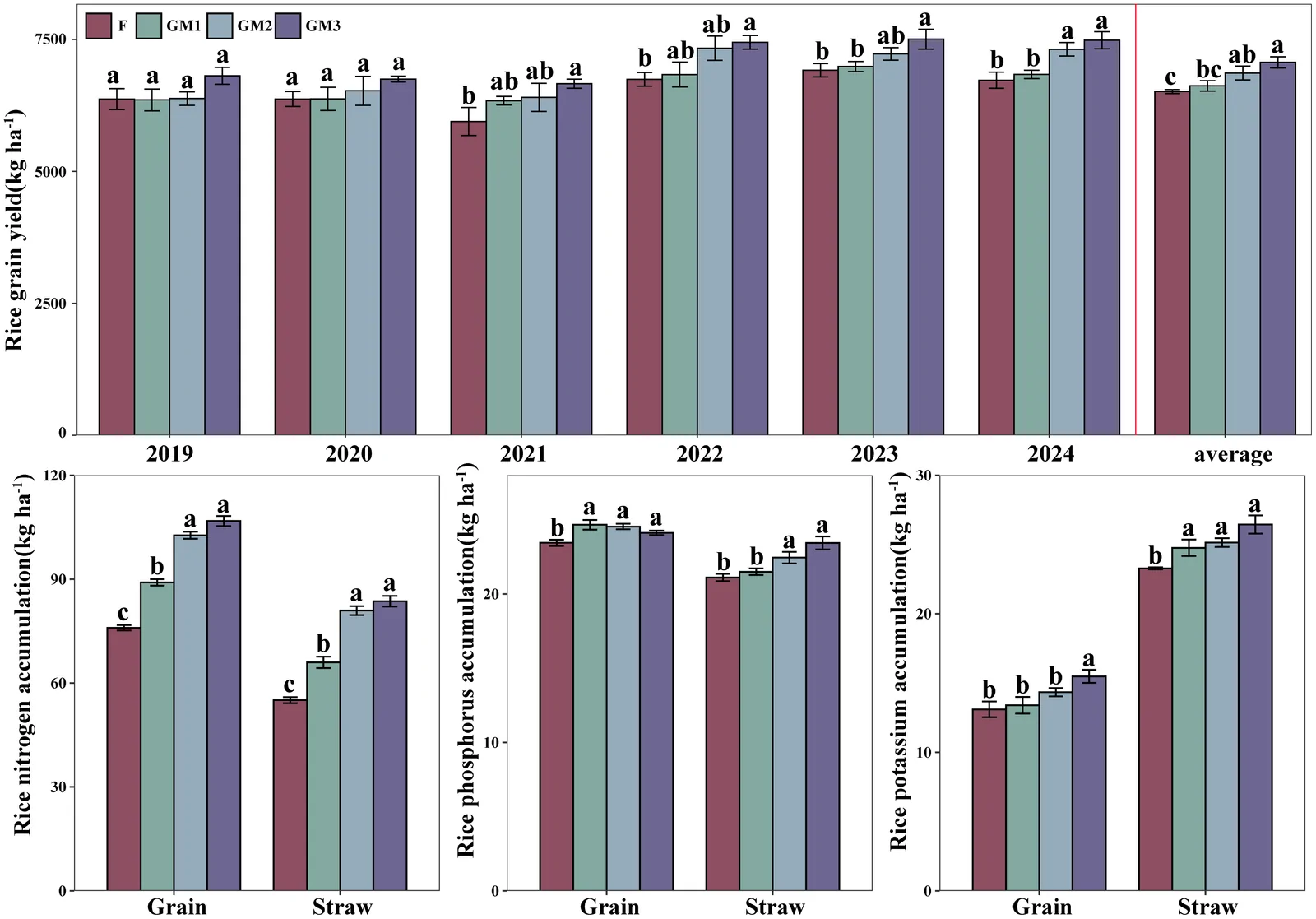
Response of rice yield and nutrients uptake to green manuring and nitrogen reduction. Bar charts reflect mean value and standard errors for each practice. The error bars on the columns represent the standard errors (SE) and the different letters indicate significant differences (*P* < 0.05) between the different treatments. F represent fallow; GM1, GM2 and GM3 represent green manuring in combination of 40% nitrogen reduction, 20% nitrogen reduction and without nitrogen reduction, respectively.

Under different GM treatments, nitrogen accumulation in both rice grain and straw were significantly higher than that in the F. Notably, grain and straw nitrogen accumulation was significantly greater in the GM2 and GM3 treatments than in the GM1 treatment. Rice grain phosphorus (P) accumulation was significantly greater in all GM treatments compared to the F treatment, with GM2 and GM3 also showing significantly higher straw P accumulation than F. For grain potassium (K) accumulation, GM3 was the highest among all treatments; meanwhile, straw K accumulation was significantly higher in all GM treatments than in the F.

### Changes in soil nitrogen pool

Differential analysis revealed that the soil total nitrogen content in the GM3 treatment was significantly lower than that in the GM2 treatment. Soil MIN contents in the GM2 and GM3 were significantly higher than those in the F, increasing by 14% and 25%, respectively (**Figure 2a**). GM2 exhibited the highest AHN content, which was significantly greater than that of other treatments, representing increases of 20%, 14% and 23% compared to F, GM1 and GM3, respectively. Further subdivision of the AHN components revealed that the contents of ASN, AAN and HUN in the GM2 were the highest, and increased by 33%, 19% and 15% compare to these in F treatment, respectively.

**Figure 2:**
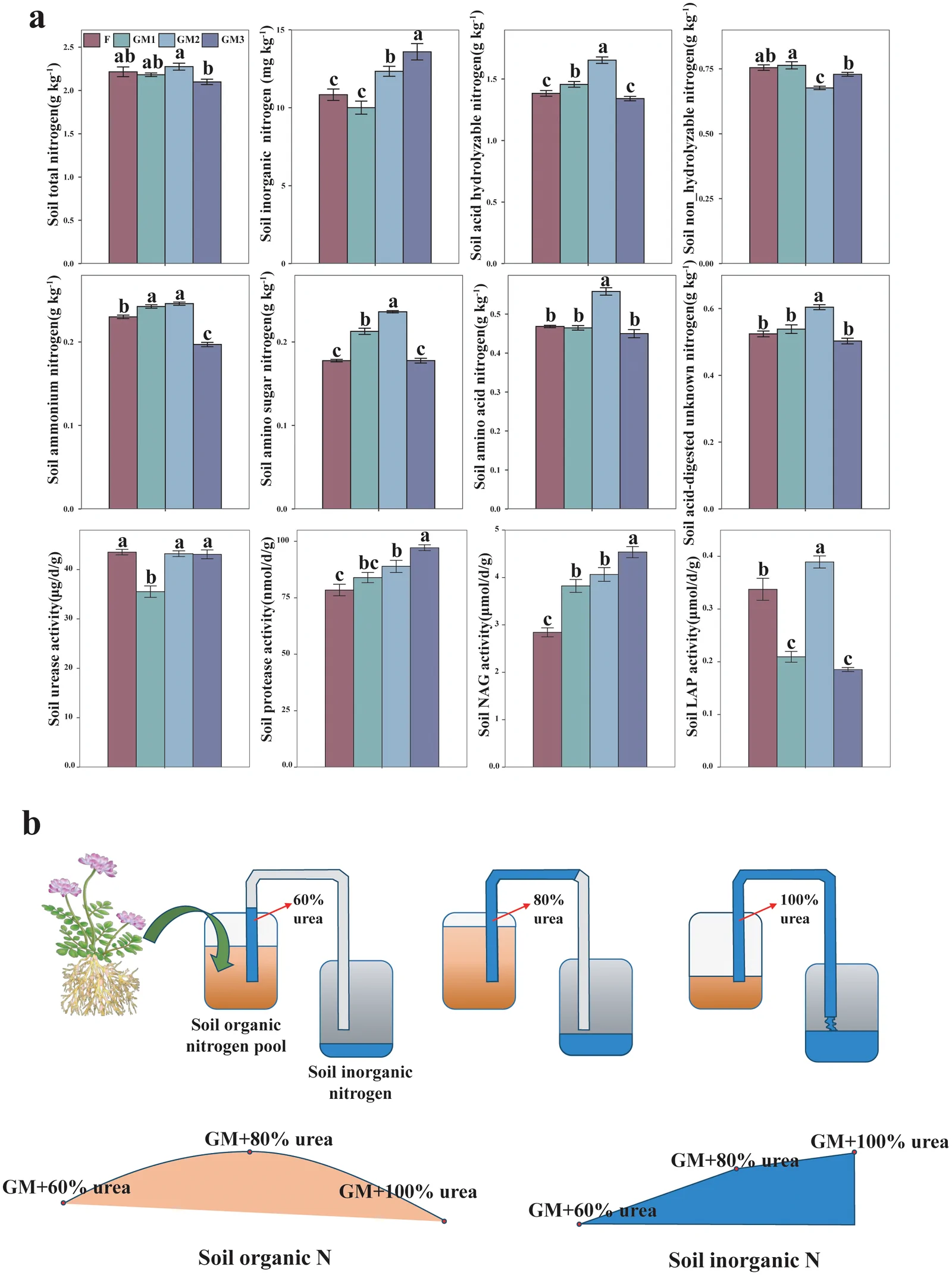
Responses of soil nitrogen fractions and organic nitrogen hydrolyzing enzyme activities to green manuring and nitrogen reduction **(a)**. Bar charts reflect mean value and standard errors for each practice. The error bars on the columns represent the standard errors (SE) and the different letters indicate significant differences (*P* < 0.05) between the different treatments. The Schematic diagram showing the changes in soil organic and inorganic nitrogen in response to different nitrogen fertilizer application rates **(b)**. The brown curved lines and blue slope areas represent the contents of soil organic nitrogen and inorganic nitrogen, respectively, across different treatments. F represent fallow; GM1, GM2 and GM3 represent green manuring in combination of 40% nitrogen reduction, 20% nitrogen reduction and without nitrogen reduction, respectively.

Urease, protease, NAG, and LAP were quantitatively analyzed to further evaluate the transformation process of organic nitrogen in the soil nitrogen pool. Under different GM treatments, soil protease and NAG enzyme activities increased with nitrogen fertilizer input (GM3 > GM2 > GM1), and all were higher than those in the F treatment. The LAP enzyme activity under the GM2 treatment was the highest, significantly exceeding that of the other treatments.

### Microbial diversity, composition and predicted functional profiles along the rice soil–root continuum

As rice nutrient acquisition depends on microbial transformation along the soil–root continuum, we characterized bacterial and fungal community structures and predicted nitrogen cycling functions across three key compartments: bulk soil, rhizosphere, and root endosphere. Treatments were main driver of bacteria and fungi composition as evidenced by a clear separation among different treatments across compartments using the CAP based on Bray-curtis distance (**Figure 3**).

**Figure 3:**
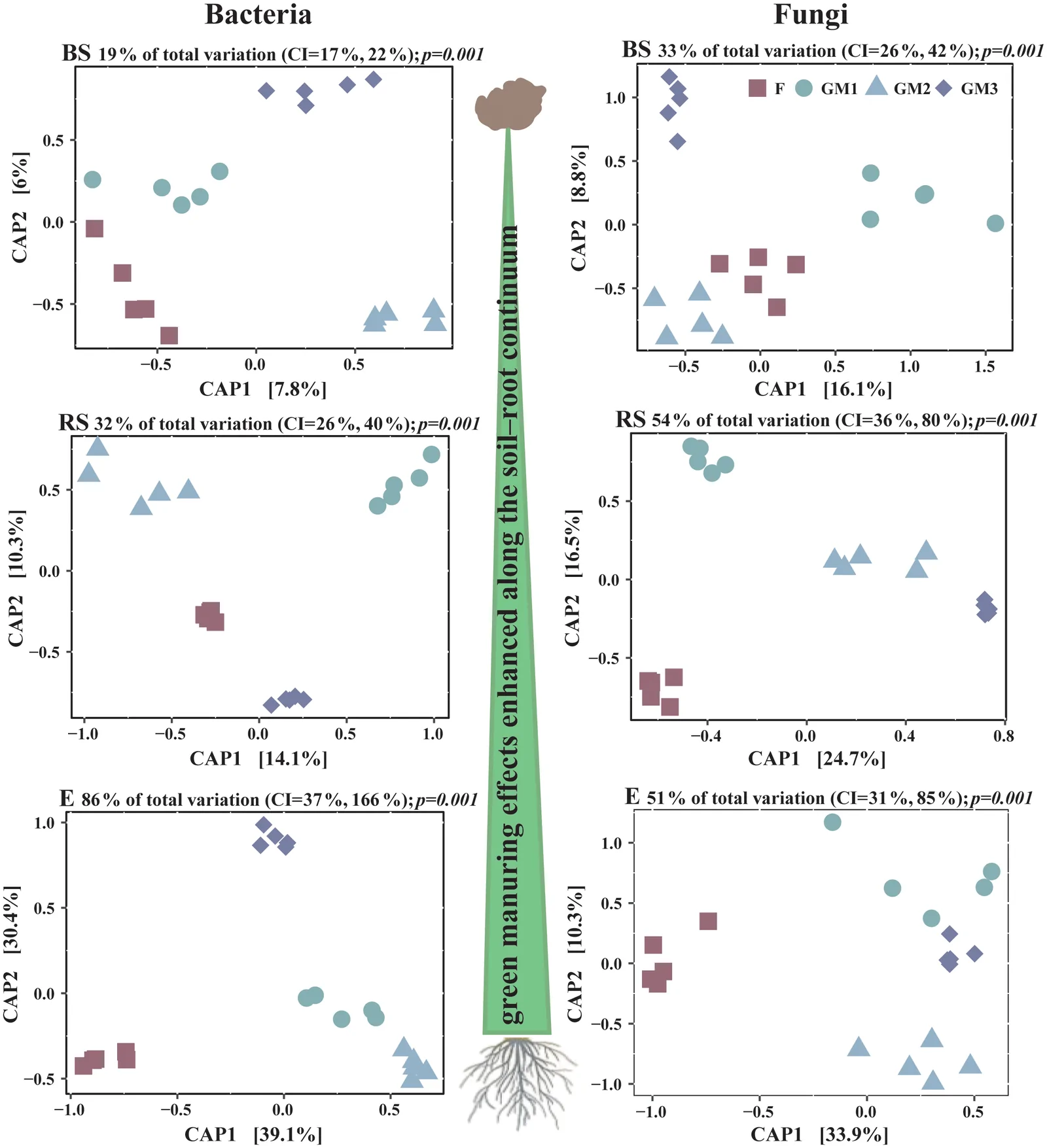
Effects of green manuring combined with reduced nitrogen fertilization on bacterial and fungal communities across different compartments. Separate CAP ordinations using Bray-curtis distance were performed for bacteria, both in bulk soil, rhizosphere soil and endosphere. CAP analyses were constrained by the factor “treatments”, and the explained fraction of the total variance is indicated above the plots (with 95% confidence interval, significance assessed with 10^4^ permutations). Percentage of variation given on each axis refers to the explained fraction of total variation. F represent fallow; GM1, GM2 and GM3 represent green manuring in combination of 40% nitrogen reduction, 20% nitrogen reduction and without nitrogen reduction, respectively. BS, bulk soil; RS, rhizosphere soil; E, root endosphere.

Results showed that the explanatory power of green manuring on microbial community variation progressively increased along the soil-rhizosphere-root continuum, from 19% to 32% to 86% for bacteria, and from 33% to 54% to 51% for fungi (**Figure 3**). The regulatory effect of green manure on microbial community structure intensifies with closer proximity to crop roots, and this pattern is stronger for bacterial than for fungal communities.

Bacterial Shannon diversity of F treatment was significantly higher in the rhizosphere than in the GM1 and GM2 treatments, while an opposite trend was observed within the root compartment (**Supplementary Figure S2**). Additionally, GM1 and GM2 exhibited significantly higher endophytic bacterial species richness. Regarding fungi, root Shannon diversity under the F treatment was significantly greater than that under all green manure treatments, and root species richness in GM1 and GM2 was significantly higher than that in GM3. These results suggest that green manure application imposes a strong directional selection on fungal communities from the root interior outward across the rhizosphere–soil continuum, thereby reducing fungal diversity. Conversely, bacterial diversity and functional species richness increase significantly within the root compartment, likely driven by enhanced carbon resources supply and nitrogen cycling.

Copiotrophic bacterial phyla, including Bacteroidota, Firmicutes, and Proteobacteria, showed progressive enrichment along the soil–root continuum under green manuring (**Supplementary Figures S1**, **S3–S8**). Differential abundance analysis further revealed compartment-specific responses to green manure treatments. In bulk soil, Bacteroidota showed a representative enrichment under GM2. In the rhizosphere, Proteobacteria were relatively enriched under GM1 and GM2, whereas distinct shifts in Actinobacteriota, Bacteroidota, and Proteobacteria were observed in the root endosphere. For fungi, Mucoromycota was preferentially enriched in the rhizosphere under green manure treatments, while Ascomycota and Rozellomycota also showed clear compartment-dependent distribution patterns along the soil–root continuum (**Supplementary Figures S1**, **S9–S14**). Rozellomycota showed distinct responses in bulk and rhizosphere soils, whereas Ascomycota was consistently enriched in the root endosphere under green manure treatments. In addition, GM2 was associated with a higher abundance of Basidiomycota in the root endosphere. Collectively, these results indicate that green manuring induced compartment-dependent shifts in dominant bacterial and fungal phyla, with the strongest compositional differentiation occurring across the soil–root continuum.

In bulk soil, those microbes inhabiting in F and GM3 showed higher functioning potential of nitrate denitrification (**Figure 4**). The effects of GM on predicted functions associated with ureolysis, nitrogen respiration, nitrogen fixation, nitrate reduction and nitrate denitrification were enhanced closed to rice root. For the rhizosphere soil, GM3 possessed the highest predicted functions related to nitrogen respiration, nitrogen fixation, nitrate reduction and nitrate denitrification. Remarkably, the GM treatments possessed higher functional potentials ureolysis, nitrogen respiration, nitrogen fixation in root endosphere, with GM3 remaining the highest. These results indicate that the root of rice shape the microbial groups with nitrogen cycling-related functions in response to different regimes of green manuring and nitrogen application.

**Figure 4:**
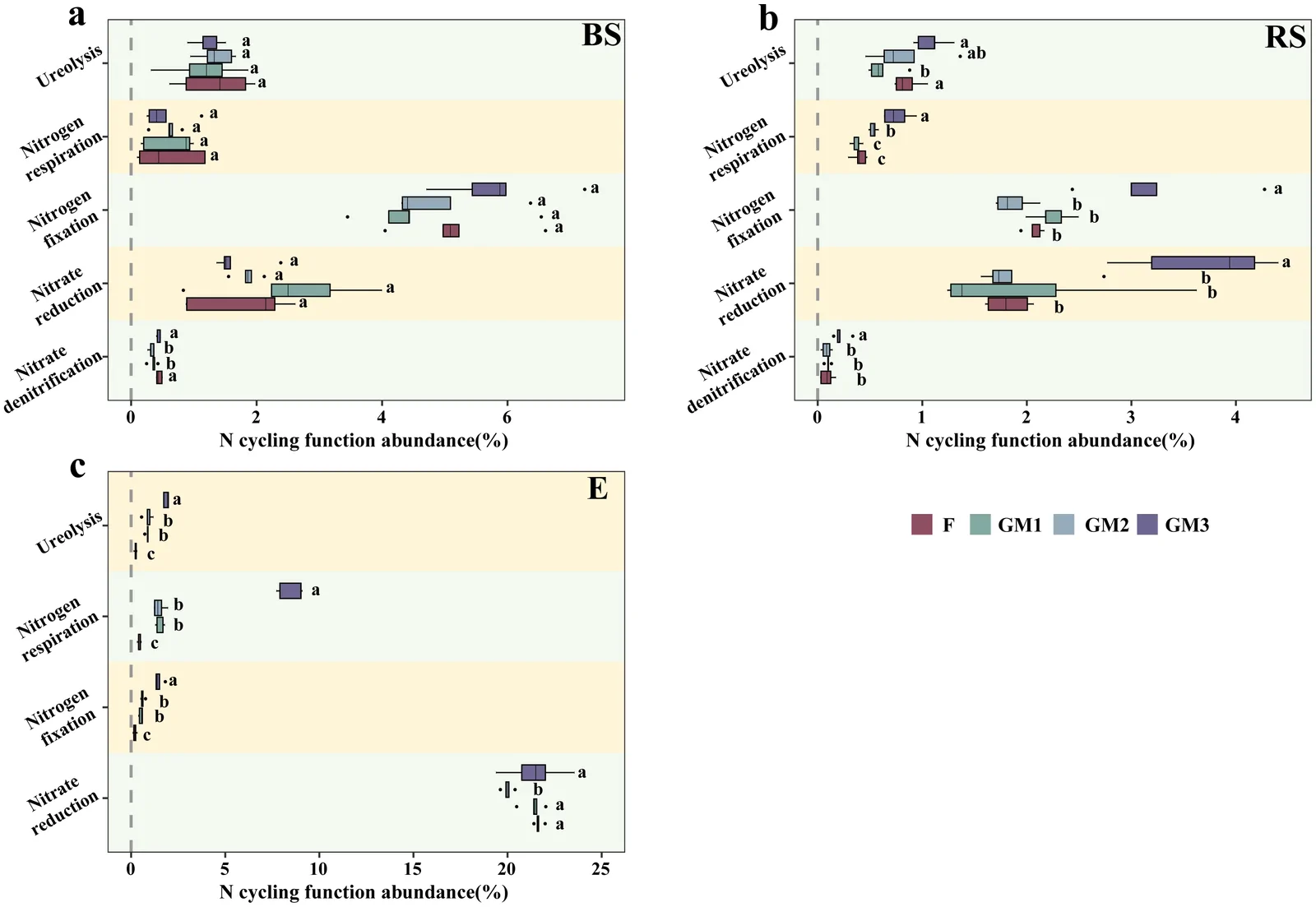
Nitrogen cycling functional profiles of microbiome across the rice soil -root continuum. **(a)** Bulk soil (BS); **(b)** rhizosphere soil (RS); **(c)** root endosphere (E). Bar charts reflect mean values and standard errors for each practice. Error bars represent standard errors (SE), and different letters indicate significant differences (P < 0.05) between treatments. F represents fallow; GM1, GM2 and GM3 represent green manuring in combination with 40% nitrogen reduction, 20% nitrogen reduction and without nitrogen reduction, respectively.

### Metabolic profiles changes in the rhizosphere driven by green manuring

To elucidate how shifts in rhizosphere metabolic profiles are linked to the reassembly of microbial community, and to uncover the resulting patterns of soil organic nitrogen pool accumulation, we identified rice rhizosphere metabolites under various treatments. The partial least squares discriminant analysis (PLS-DA) showed that the metabolites compositions of various treatments were significantly separated (R = 0.74, *P* = 0.001). Notably, amino acids, fatty acids, and carbohydrates were the dominant groups across various treatments, collectively accounting for more than 25% of the total metabolome (**Supplementary Figure S18**). Significant differences were observed in the enrichment patterns of these three metabolites across different treatments (**Supplementary Figures S24–26**).

ANOVA analysis was conducted to compare the profiles of the top 10 most abundant metabolites across different treatments. At subclass level, it was found that abundances of amino acids in GM2 were significantly higher than those in both F and GM3. The highest concentration of androstane steroids, eicosanoids, fatty acids and retinoids were observed in GM1, which significantly higher than that in F and GM2.

Compared with the F treatment, 232 metabolites were significantly enriched commonly across all the GM treatments (**Figures 5d, e**). Specifically, the number of uniquely enriched metabolites in the GM1, GM2 and GM3 treatments was 278, 281 and 139, respectively (**Supplementary Figure S19**). In both GM1 and GM2 treatments, the most highly enriched individual metabolites exhibited a consistent occurrence pattern, including melilotoside, Gly-Trp, and D-Synephrine et.c. A total of 102 metabolites were uniquely enriched in GM2, predominantly organic acids, with L-Leucine and L-Isoleucine exhibiting the highest abundances. In contrast, GM1 and GM3 contained 106 and 19 metabolites, respectively, where the most abundant compounds were 17Alpha-Methyltestosterone and Dodecanoic Acid, both of which primarily belonged to the lipid category (**Figure 6c**). Analysis of shared and uniquely enriched metabolites across different green manure treatments identified 93 metabolites common to all GM treatments. These metabolites were predominantly various organic acids, with N6-Acetyl-L-Lysine being the most abundant. The metabolites commonly enriched across treatments were interpreted as resulting from adaptive changes in the metabolic profile of the rice rhizosphere in response to green manuring.

**Figure 5:**
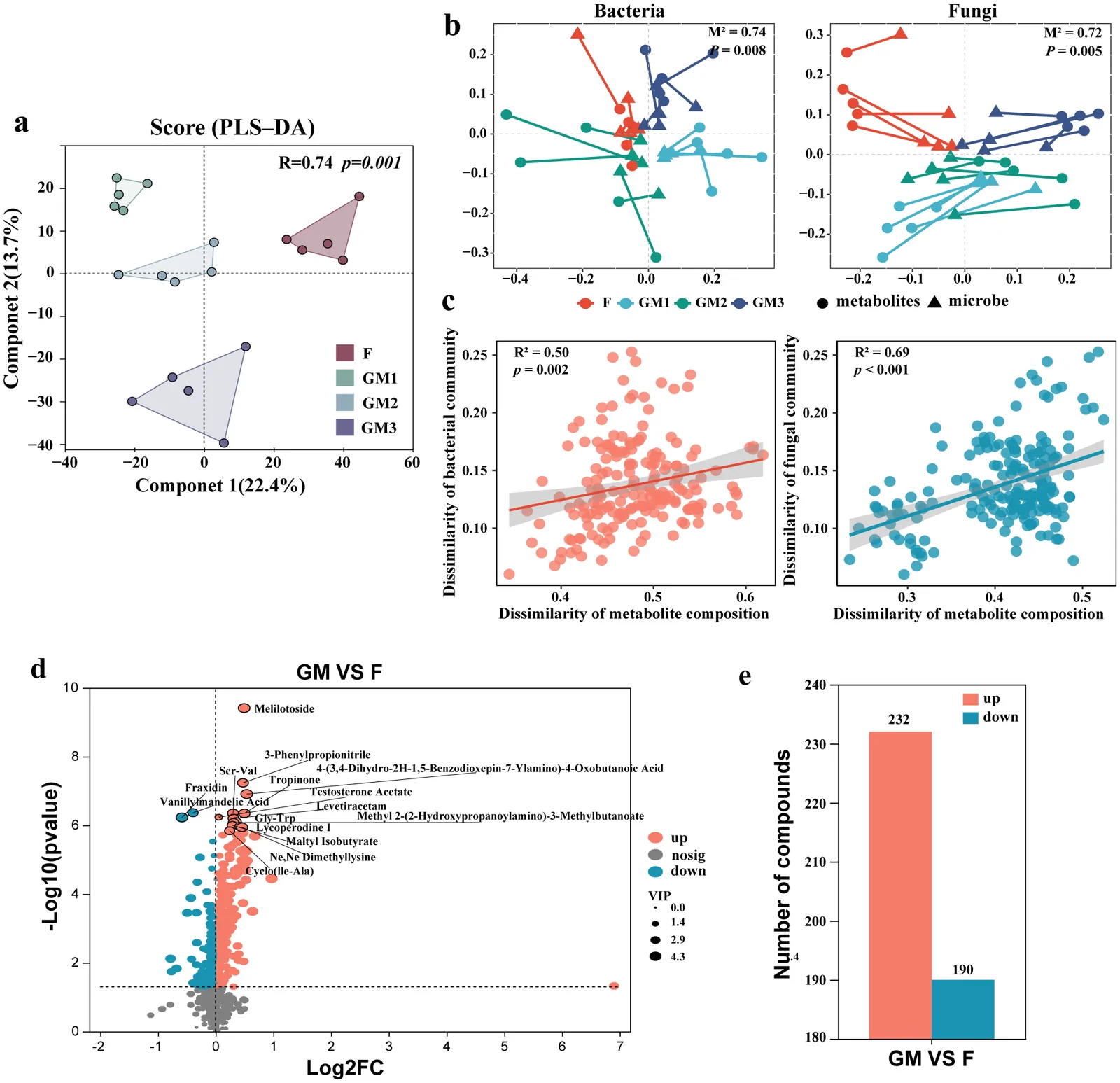
Partial least squares-discriminant analysis (PLS-DA) of the rhizosphere soil metabolome **(a)**. Procrustes analysis and Mantel test evaluating the relationships between rhizosphere metabolite composition and functional microbial population **(b)**. The correlations between the dissimilarity of metabolite composition and that of microbial community (based on Bray-Crutis distance, **(c)**. And enrichment and depleted patterns of rice rhizosphere metabolites between green manuring and fallow **(d, e)**. F represent fallow; GM1, GM2 and GM3 represent green manuring in combination of 40% nitrogen reduction, 20% nitrogen reduction and without nitrogen reduction, respectively. BS, bulk soil; RS, rhizosphere soil; E, root endosphere.

**Figure 6:**
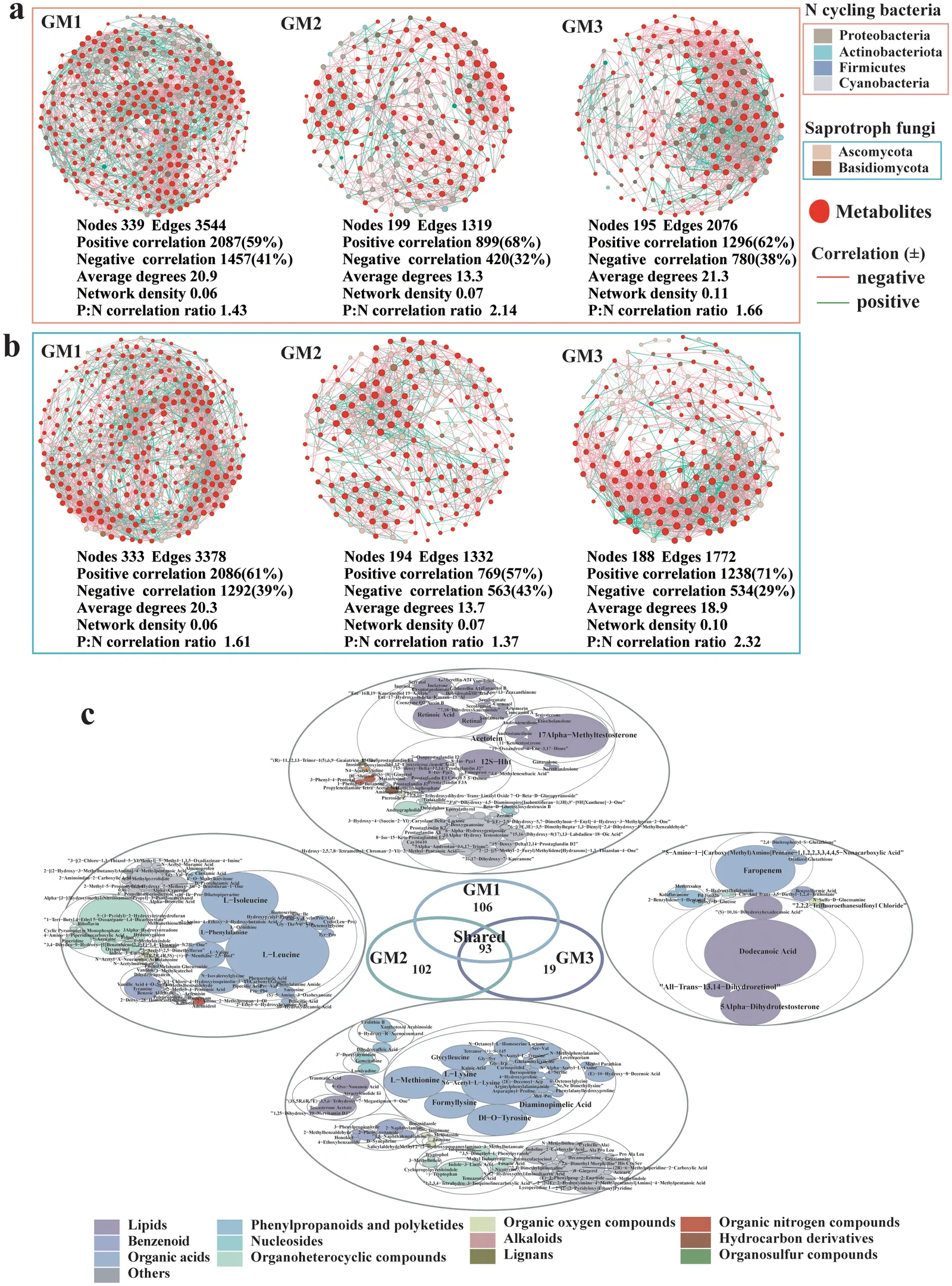
Co-occurrence network of rhizosphere metabolites and N cycling related bacteria **(a)** and saprotroph fungi **(b)**. In the network, the connection stands for a strong (Spearman's r>0.85) and significant (P < 0.05) correlation. Node color represents N cycling related bacteria, saprotroph fungi and metabolites (red). Green lines indicate positive interactions, and red lines indicate negative interactions. Differences in relative abundance of practice exclusive and shared rhizosphere soil enriched metabolites level **(c)**. The bubble sizes represent the relative abundance of metabolites. The largest circles represent super-class level, and the inner circles represent class, sub-class, and individual metabolite. Metabolites are colored according to super-class level. GM1, GM2 and GM3 represent green manuring in combination of 40% nitrogen reduction, 20% nitrogen reduction and without nitrogen reduction, respectively.

### Association analysis between rhizosphere metabolomic shifts and microbial communities in rice

Procrustes analysis was performed to investigate the association between the altered soil-root continuum microbiome and rice rhizosphere metabolites (**Figures 5b**; **Supplementary Figures 16, 17**). Results showed a strong correlation between microbiome of rhizosphere soil and metabolome profiles (bacteria: *P* = 0.008, M^2^ = 0.74; fungi: *P* = 0.005, M^2^ = 0.72; **Figure 5b**). And a significant correlation between fungal community in rice endosphere and rhizosphere metabolome were observed (*P* < 0.001, M^2^ = 0.57; **Supplementary Figure S17**). Then the PLS-DA axis1 of rhizosphere soil metabolites, representing the metabolite composition, was used to evaluate its correlation with the rhizosphere soil microbial community. It was found that the dissimilarity of bacterial (R = 0.50, *P* = 0.002) and fungal (R = 0.69, *P* < 0.001) communities were significantly correlated with metabolite composition in rhizosphere soil (**Figure 5c**). These findings suggest that rice metabolic activities in the rhizosphere can effectively drive microbial assembly within the corresponding niche.

Nitrogen-cycling related bacteria and saprotrophic fungi were identified through FAPROTAX and FUNGuild, followed by co-occurrence network analysis with green manuring enriched metabolites to evaluate the interaction landscape between corresponding functional microbial communities and metabolites across different GM treatments (**Figures 6a, b**). Multiple topological metrics indicated that the networks complexities of GM1 was significantly different from those of GM2 and GM3. The metabolite-bacterial and metabolite-fungal co-occurrence networks in GM1 consisted of 339 nodes with 3,544 edges and 333 nodes with 3,378 edges, respectively. These values were markedly higher than those observed in GM2 (bacteria: 199 nodes, 1,319 edges; fungi: 194 nodes, 1,332 edges) and GM3 (bacteria: 195 nodes, 2,076 edges; fungi: 188 nodes, 1,772 edges). The network average degrees for bacteria and fungi in GM2 were 13.3 and 13.7, respectively, both of which were lower than those observed in GM1 and GM3. The lower network complexity in GM2, characterized by fewer nodes and edges, may reflect reduced functional redundancy and greater metabolic specialization within the microbial community, potentially contributing to the observed efficiency in organic nitrogen transformation.

For the metabolites enriched across different GM treatments, we performed machine learning using a random forest model to identify potential components associated with the accumulation of soil organic nitrogen and inorganic nitrogen. Random forest models were concurrently built on distinct datasets to assess the explanatory power of GM1, GM2, and GM3 specific enriched metabolites, as well as shared metabolites, for AHN and MIN. The explanatory power values were 30.04 for GM1, 65.75 for GM2, 1.53 for GM3, and 26.9 for the shared metabolites. For MIN, the explanatory power of random forest models constructed with treatment GM1, 2, 3 metabolites were 19.53, 0.11, and 42.87, respectively, while that of the shared metabolites was 1.77. Through model optimization and selection, GM2 and GM3 specific enriched metabolites were identified as the metabolites with the greatest potential to drive AHN and MIN accumulation, respectively. A total of 16 metabolites made significant contributions to AHN accumulation (**Figure 7a**), and four metabolites made significant contributions to MIN accumulation (**Figure 7b**). Among these, 14 and 4 metabolites showed positive contributions to AHN and MIN accumulation, respectively (**Supplementary Figures S27–28**), with CThzMOI and GlcN-S being the strongest effectors, respectively. The metabolites that significantly contributed to AHN and MIN accumulation exhibited the highest abundances in GM2 and GM3 treatments, respectively, reflecting their potential to drive the accumulation of the corresponding nitrogen component (**Figures 7e, f**).

**Figure 7:**
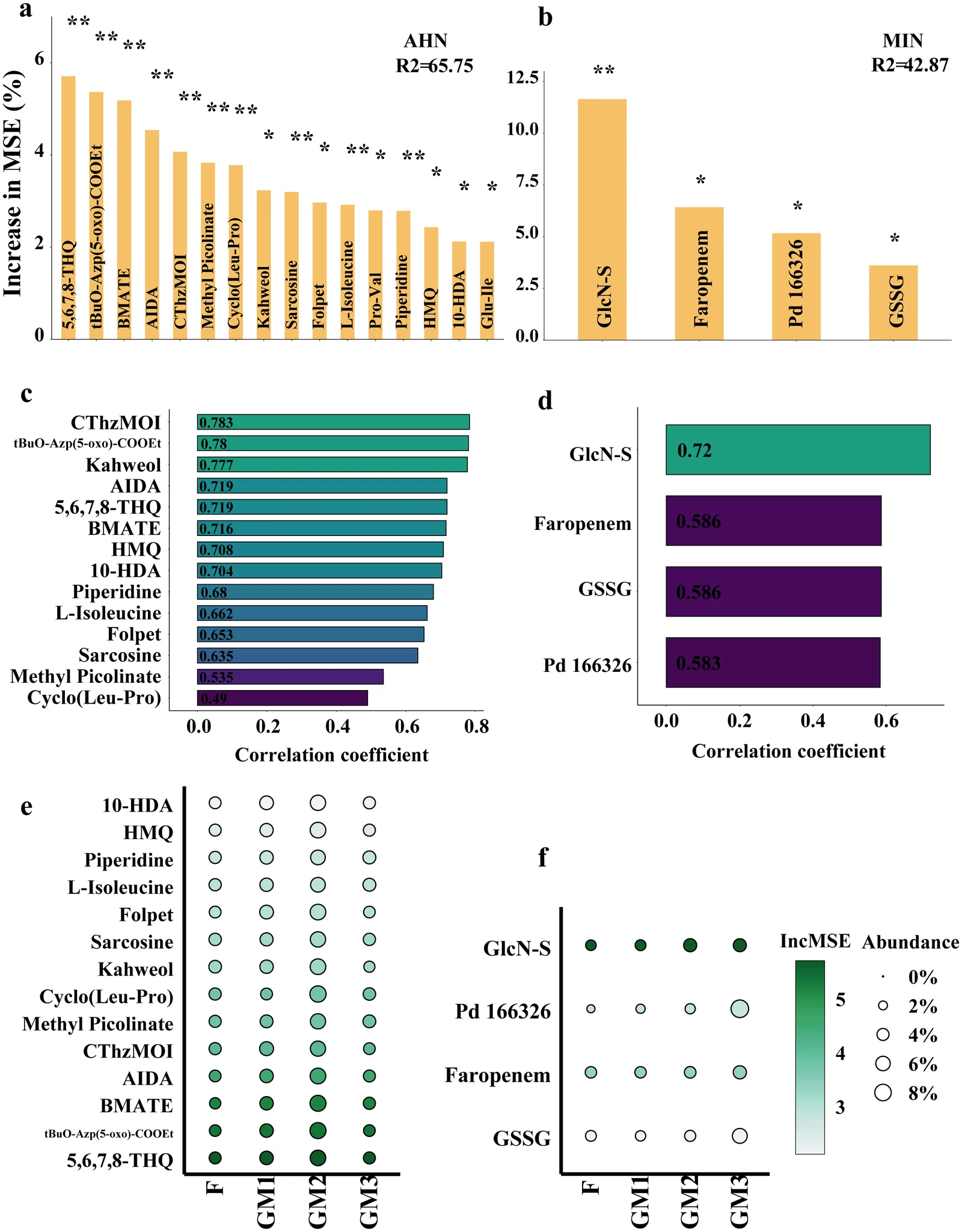
Random forest-based machine learning to identify key green manuring-enriched metabolites contributing significantly to AHN and MIN accumulation **(a–f)**. Abbreviations in **(a–f)** denote the individual metabolites, HMQ:2-(hydroxymethyl)-4(3H)-quinazolinone;CThzMOI:3-[(2-Chloro-1,3-Thiazol-5-Yl)Methyl]-5-Methyl-1,3,5-Oxadiazinan-4-Imine; AIDA:2-Aminoindan-2-Carboxylic Acid; BMATE:2-[Benzyl (Methyl) Amino] Ethyl Thiophene-2-Carboxylate; tBuO-Azp(5-oxo)-COOEt: 1-Tert-Butyl-4-Ethyl-5-Oxoazepane-1,4-Dicarboxylate; 5,6,7,8-THQ: 5,6,7,8-Tetrahydro-2-quinolone;10-HAD: 10-Hydroxydecanoic Acid; GlcN-S: N-Sulfo-D-Glucosamine; GSSG: Oxidized Glutathione. Asterisks (** and *) indicate significance at probability levels of *P* < 0.01 and 0.05. GM1, GM2 and GM3 represent green manuring in combination of 40% nitrogen reduction, 20% nitrogen reduction and without nitrogen reduction, respectively.

To further elucidate how rice root activity drives microbial taxa in the transformation and accumulation of organic and inorganic nitrogen, we conducted a correlation analysis between the metabolite drivers identified by machine learning models and bacterial and fungal taxa. Heatmaps showed that at the highest resolvable classifiable taxonomic level (class level), the bacterial classes *Gammaproteobacteria*, *Negativicutes*, and *Syntrophomonadia* exhibited significant positive correlations with more than five AHN accumulation- associated metabolites (**Figure 8a**). Among fungi, *Sordariomycetes* and *Endogonomycetes* showed significant positive correlations with three or more AHN accumulation-associated metabolites (**Figure 8b**). Among bacterial taxa, *norank_p_NB1−j*, *OLB14*, *Dadabacteriia*, *Desulfuromonadia*, *SJA-28*, *Thermodesulfovibrionia*, *Desulfobacteria*, and *norank_p_Sva0485* were significantly and positively associated with at least three metabolites implicated in associated with MIN accumulation (**Figure 8c**). In contrast, only *norank_p_Rozellomycotina* among fungi exhibited significant positive correlations with the entire set of such metabolites (**Figure 8d**).

**Figure 8:**
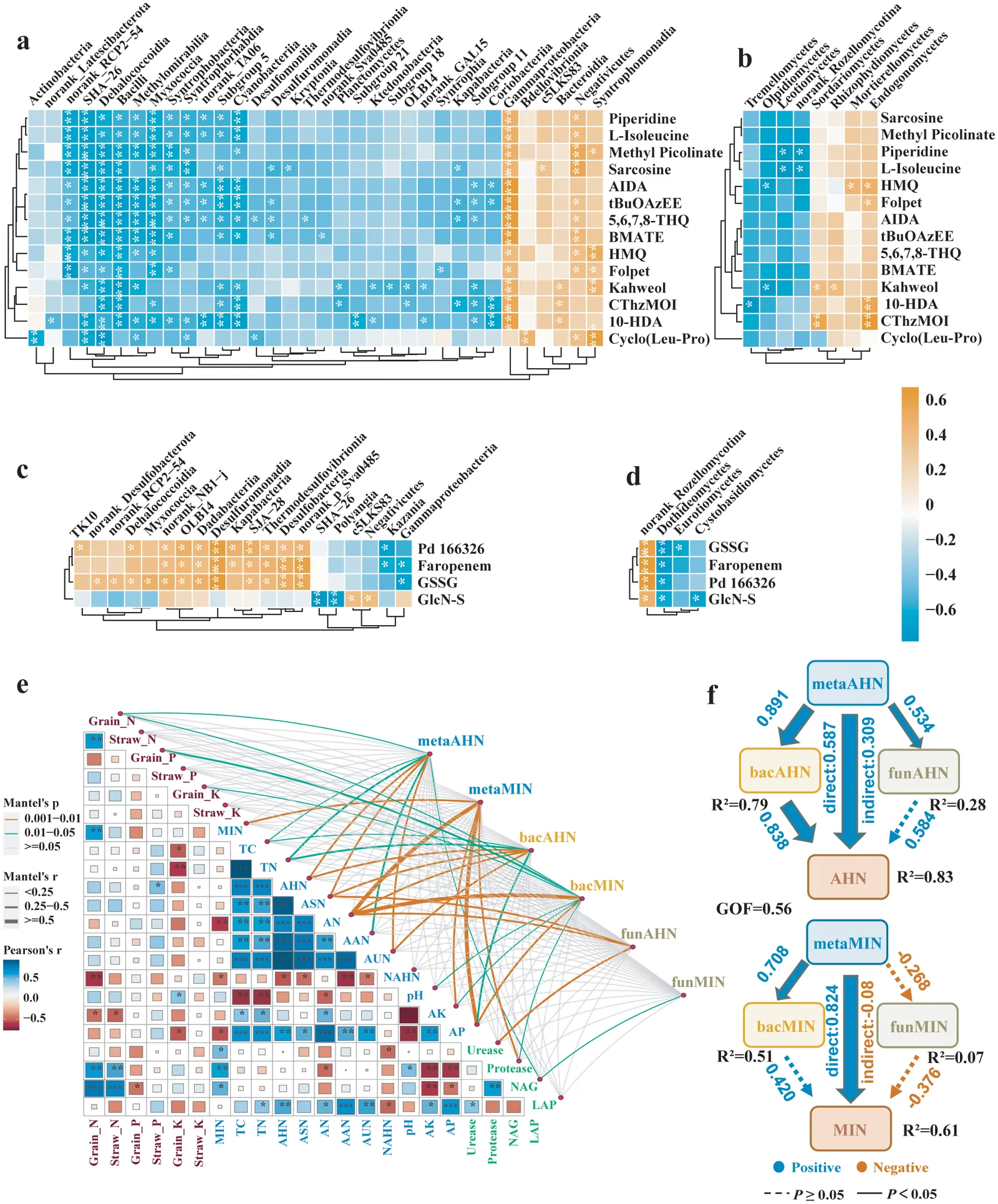
Correlation and Mantel analysis of nitrogen-cycling elements. Correlation heatmaps between nitrogen-cycling associated metabolites and nitrogen-cycling bacteria as well as saprotrophic fungi **(a–d)**. Asterisks (** and *) indicate significance at probability levels of *P* < 0.01 and 0.05. Mantel test revealing the relationships of nitrogen-cycling-related metabolites and microorganisms with soil and rice properties **(e)**. PLS-PM reveal the direct and indirect effects of the metabolites and microbes on soil AHN and MIN accumulation **(f)**.

### Rhizosphere metabolite–microbe associations underpin soil nitrogen accumulation

Mantel analysis was performed to integrate the metabolites and microbes potentially driving organic and inorganic nitrogen accumulation, and to link them with soil properties and rice nutrient contents. Metabolites that associated with AHN accumulation (metaAHN) showed significant positive correlations with soil nitrogen fractions (TN, AHN, ASN, AUN) and rice grain nitrogen content (**Figure 8e**). In addition to promoting MIN accumulation, metabolites that associated with MIN accumulation (metaMIN) was also significantly positively associated with AN, AK, and AP contents. Bacteria capable of promoting AHN accumulation (bacAHN) enhanced the accumulation of all organic nitrogen components except for AHN. Bacteria capable of enhancing MIN accumulation (BacMIN) were tightly coupled with grain N, P, and K accumulation; furthermore, this bacterial group also drove the enrichment of soil AN, AK, and AP.

The AHN accumulation facilitating fungi (funAHN) exhibited significant correlations with both ASN and AN. In contrast, no promoting effects on plant or soil nutrient accumulation were observed for fungi that enhance MIN accumulation (funMIN). Among the soil enzymes, urease was significantly correlated with funAHN, protease was significantly associated with both metaMIN and bacMIN, and funMIN showed a significant correlation with NAG. These results suggest that the accumulation of the corresponding soil nutrients was co-driven by metabolites and microbes.

Results of PLS-PM showed that metaAHN was significantly and positively correlated with the bacAHN (e=0.891, *P* < 0.001) and funAHN (e=0.534, *P* < 0.05) (**Figure 8f**). The accumulation of AHN was significantly and positively correlated with bacAHN (e = 0.838, *P* < 0.05), while no significant correlation was observed between funAHN and AHN accumulation (e = 0.584, *P*>0.05). MetaMIN had a notable association with bacMIN (e = 0.708, *P* < 0.05). The bacMIN and funMIN had positive and negative effects on MIN accumulation, respectively, but neither was significant. Additionally, metaAHN was positively associated with AHN accumulation via both direct (e = 0.587) and indirect (e = 0.309) effects. In contrast, while the direct effect of metaMIN on MIN accumulation was positive and significant (e = 0.824 *P* < 0.01), it was accompanied by a slight negative effect (e = -0.08, *P*>0.05).

## Discussion

Green manure combined with moderate nitrogen reduction should be viewed not merely as a fertilizer-saving option, but as a practical restoration pathway for degraded croplands. Across field and long-term studies, this integrated strategy enhances soil organic carbon, nitrogen availability, microbial biomass, enzyme activity (**Figure 9**), and aggregate stability, indicating recovery of both soil fertility and biological function (Hou et al., 2022; Qiu et al., 2025). At the same time, moderate reductions in synthetic N commonly around 20% are often sufficient to maintain grain yield, nitrogen use efficiency, and water productivity when supported by green manure inputs, whereas more severe N cuts are less reliable (Wang P et al., 2024). This gives the findings clear practical significance: the practice offers farmers a realistic way to restore degraded cropland function, reduce fertilizer dependence, and lower environmental losses without compromising production, making it a promising strategy for sustainable crop production.

**Figure 9:**
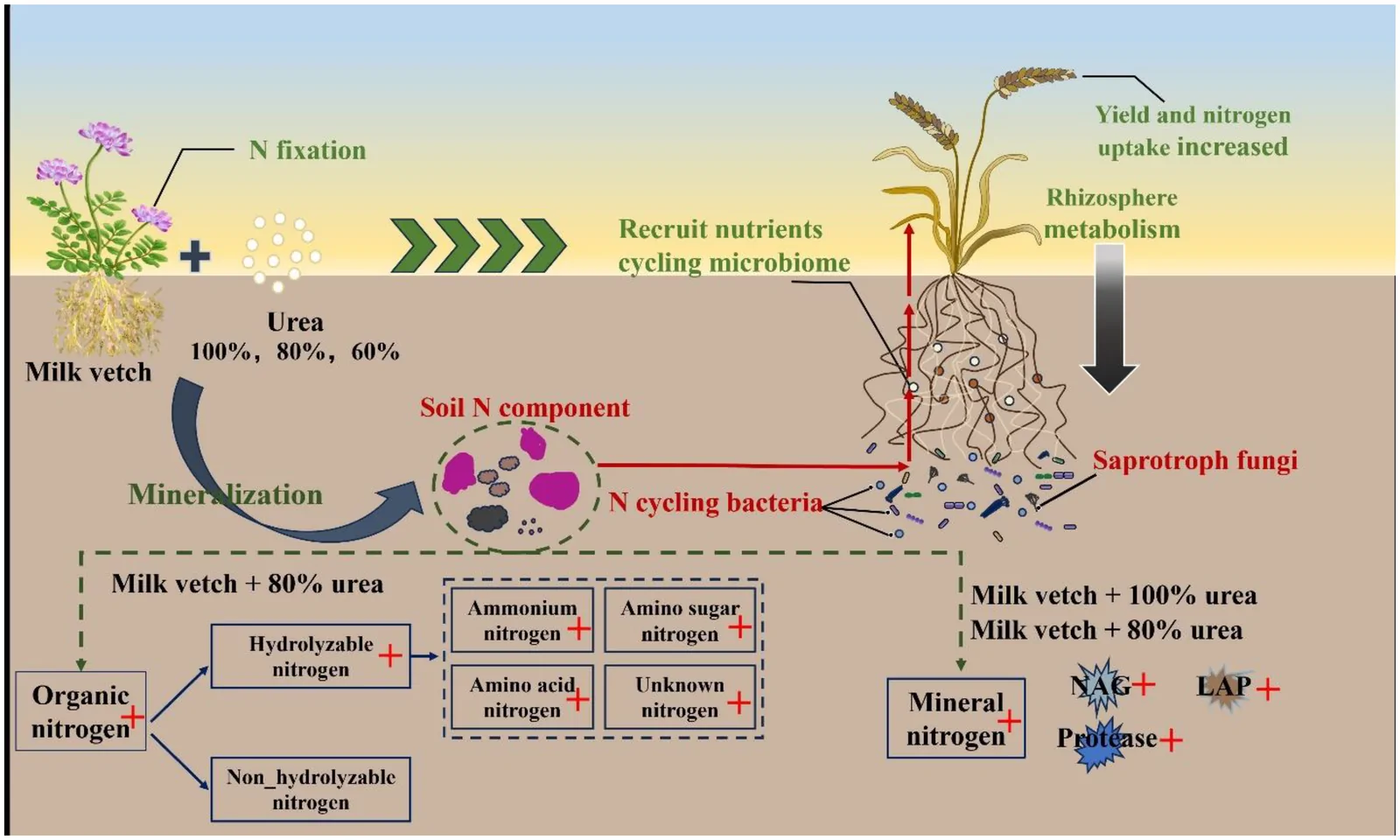
Green manuring combined with reduced nitrogen fertilization promoted coordinated rhizosphere metabolite–microbiome interactions that simultaneously sustained rice productivity and improved soil nitrogen pool stability.

### Green manuring combined with nitrogen reduction can achieve stable crop yields and expand the soil organic nitrogen pool

Based on a six-year field experiment, this study revealed that green manure application combined with nitrogen fertilizer reduction has a long-term effect on rice yield and soil nitrogen pool succession. Over the past few decades, the global rice yield has increased by an average of 25% due to the application of green manure (Xia et al., 2025). The contribution of leguminous green manure, such as milk vetch, to rice yield is attributed to the substantial organic nitrogen input into the agroecosystem, as well as the enhanced turnover of nitrogen fractions in the soil and improved nitrogen uptake by rice (Hou et al., 2022; Li et al., 2022). Green manuring combined with 20% (GM2) and 40% (GM1) reduction of nitrogen fertilizer can maintain rice yield while significantly expanding the soil organic nitrogen pool. In contrast, green manuring without nitrogen reduction (GM3), although increasing yield, leads to a significant decline in the soil organic nitrogen components (**Figure 2b**). This highlights the primary trade-off among crop yield, soil health, and nutrient input: while excessive application of chemical nitrogen fertilizer may increase yield in the short term, it can come at the cost of depleting soil organic matter, which is detrimental to soil sustainability in the long run. Notably, GM2 resulted in a significantly higher grain yield and grain nitrogen accumulation than GM1 by 2024, suggesting that a 20% reduction in chemical nitrogen fertilizer application may represent an optimal strategy for synergistically enhancing crop yield and soil fertility.

Soil organic nitrogen components are influenced by agricultural practices and serve as important indicators for evaluating soil nitrogen supply to crops (Giannetta et al., 2018; Yang et al., 2018). In this study, green manure residues represent the dominant organic nitrogen input, which comprises the largest nitrogen stock in soil organic matter (Jan et al., 2009). Thus, the breakdown of proteins to amino acids by extracellular proteases is central to plant nitrogen availability and is proposed to be the rate limiting step of nitrogen cycling in soil (Sieradzki et al., 2023; Weintraub and Schimel, 2005). The variation in protease and LAP enzyme activities among treatments was associated with the hydrolytic conversion of macromolecular organic nitrogen in green manure residues and the accumulation of small-molecular organic nitrogen in soil (Sieradzki et al., 2023). GM2 significantly enhances the activity of protease and LAP, effectively catalyzing the hydrolysis of peptides and proteins in the soil into amino acids. Among the various components of soil organic nitrogen pool, the contents of soil ASN and AAN in the GM2 were significantly higher than those in the other treatments. Soil ASN is primarily derived from the continuous accumulation of microbial cell wall residues. Due to its relatively stable structure and resistance to rapid decomposition, it is considered a key component of the stable soil organic nitrogen. AAN is a relatively active organic nitrogen component and an important intermediate in the soil nitrogen cycle because it is readily mineralized into ammonium nitrogen, which is then absorbed by plants. Collectively, this indicates that GM2 optimized the soil nitrogen pool by simultaneously enlarging both the stable and the relatively labile organic nitrogen components.

In addition, the activities of soil protease and N-acetyl-β-D-glucosaminidase (NAG) were significantly increased in GM3, indicating that high exogenous nitrogen input accelerated the mineralization of organic nitrogen to support the increase in crop yield (Yu et al., 2026). This may enhance nutrient uptake by the current crop, but at the same time increase the risk of nitrogen loss (Zhang et al., 2015). The combination of green manuring with high rates nitrogen fertilizer application induced a positive priming effect through the “microbial N pump” mechanism (Lei et al., 2026; Wang et al., 2026), as evidenced by the significant reductions in soil TC and organic nitrogen in the GM3. The practice which accelerates soil organic matter decomposition, may lead to a net loss of the soil organic nitrogen pool in the long term (Xu et al., 2023). Therefore, from the perspective of sustainable soil management, the strategy of green manure incorporation and nitrogen fertilizer input reduction (particularly a 20% reduction) obtains a better balance between crop yield and soil health.

### Reassembly of microbial communities in the soil-root continuum and its response to green manure and nitrogen reduction

The spatial differentiation of microbial community structure along the soil-root continuum represents the crop’s response to green manuring, thereby sustaining rhizosphere nutrient mobilization and acquisition (Liang et al., 2026). The intensity of crop responses to different GM treatment gradually increased from the bulk soil to the root endosphere. This is attributed to the explanatory power of treatment for microbial community variation progressively increased along the continuum from bulk soil to rhizosphere soil to the root compartment, rising from 19% to 86% for bacteria and from 33% to 51% for fungi (**Figure 3**). Which implies that crops stimulate and suppress distinct microbial community members through root metabolic activities, thereby shaping functionally characteristic microbial communities from the inside out to achieve enhanced its fitness (Rogier F. Doornbos et al., 2012). In contrast to fungi, this response pattern was more pronounced in bacterial communities, indicating that bacteria possess greater flexibility in adapting to different nitrogen management regimes under the influence of crop root activities. The decline in bacterial and fungal diversity observed in both bulk and rhizosphere soils is attributed to nutrient enrichment resulting from leguminous green manure incorporation and nitrogen management (Hoek et al., 2016; Hu et al., 2024). The enrichment of nitrogen and carbon sources may shift microbial interactions from mutualism to competition, thus favoring the dominance of efficient and competitive microbial species, ultimately leading to a reduction in community diversity (Hoek et al., 2016). In the root endosphere, bacterial alpha diversity under green manuring increased significantly, in contrast to fungi. Further suggests that endophytic bacteria adopt a more adaptive overall growth strategy through broad-spectrum resource utilization (Uribe-Acosta et al., 2025).

Previous studies have found that under anthropogenic nutrient enrichment, copiotrophic taxa become dominant in the soil, while leads to oligotrophic taxa disappear (Banerjee et al., 2024; Peng et al., 2024). Our findings also showed that the overall community composition under green manuring, copiotrophic bacterial species, including Bacteroidota, Firmicutes, and Proteobacteria, gradually enriched along the soil-root continuum. This also aligns with the findings of a study that indicated a pattern of carbon substrate content distribution in root exudates favoring the growth and colonization of copiotrophic bacteria in the rhizosphere region (Loo et al., 2024). And for fungal community, Ascomycota exhibited a similar enrichment pattern. This phylum contains a variety of saprophytic microbes that play an important role in the decomposition of organic matter and nutrient mineralization, thus participate in the mineralization of green manure residues (Zhang et al., 2025).

There are studies suggest that microbial functional traits are more representative than taxonomy in response to changes in nutrients (Liu et al., 2023; Peng et al., 2025). Functional prediction revealed the distinct niche differentiation in nitrogen cycling functions under GM treatments. There are contradictory findings among studies regarding the response of nitrogen-cycling functional potentials to nitrogen fertilizer inputs. For example, the abundance of nitrogen fixation-related genes (nifH and nifA) in soil decreases following inorganic nitrogen fertilization. Conversely, the application of ammonium nitrogen fertilizer stimulates soil nitrification by increasing the abundance of amoA and amoB genes. In our study, the GM3 exhibited the highest gene abundance of nitrogen respiration, nitrogen fixation, nitrate reduction, and nitrate denitrification functions in the rhizosphere soil. For the root endosphere, GM treatments (particularly GM3) still maintained high ureolysis, nitrogen respiration, and nitrogen fixation potential. This indicates that, under green manuring, nitrogen input reduction stimulated the nitrogen transformation activity of microbiome in rhizosphere, which corresponded to the soil inorganic nitrogen increase of the GM2 and GM3. Moreover, endophytic microbes may serve as a functional reservoir, playing a critical role in nitrogen supply during the peak nutrient demand period of crop (Salas-González et al., 2021).

### Rhizosphere metabolite shifts were associated with microbial community reassembly and corporation with the expansion of the soil labile organic nitrogen pool

Plant roots selectively recruit the associated microbial communities through the release of root exudates, the complex mixture organic compounds which mainly includes sugars, amino acids, and secondary metabolites, thereby adapting to nutrient fluctuations and biotic and abiotic stresses (Fröschel et al., 2021; Hu et al., 2018; Huang et al., 2019). The present study reveals that rice rhizosphere metabolic profiles under green manuring differed significantly from that under fallow, and that changes in the rhizosphere metabolic profiles exhibited strong synchrony with microbial community composition. These findings support the existence of a rhizosphere metabolite-microbe coupled regulation, through which rice selectively recruits specific functional microbes by modulating its rhizosphere metabolite profiles in response to various green manuring and nitrogen reduction strategies (Pereira-Mora et al., 2022; Wang et al., 2023).

A total of 320 unique rhizosphere enriched metabolites were identified across the green manuring treatments, among which amino acids, fatty acids, and carbohydrates are the predominant components, collectively accounting for over 25% of the total metabolites. To verify the interactions between metabolites and functional microbes, nitrogen-cycling related bacteria and saprotrophic fungal taxa were selected for co-occurrence network construct. We found that the metabolite-microbe network complexity was highest in the GM1 treatment (40% nitrogen reduction), whereas it was significantly lower in the GM2 and GM3 treatments (**Figures 6a, b**). Under insufficient exogenous nitrogen fertilizer input (GM1), crops enhance the broad-spectrum associations between rhizosphere metabolites and microorganisms to improve nitrogen availability in the rhizosphere, representing a broad-spectrum resource acquisition strategy (Jiang et al., 2024). In contrast, under relatively sufficient nitrogen input (GM2, GM3), the associations between rhizosphere metabolites and nitrogen-cycling microorganisms became more specialized, focusing on a few efficient nitrogen transformation pathways (Baker et al., 2024; Hao et al., 2025). The simplified correlation between nitrogen-cycling related bacteria and enriched metabolites, supporting the specialized functional reinforcement (**Figure 6a**).

Notably, melilotoside was significantly enriched in all GM treatments. Released into the soil during the decomposition of green manure residues, melilotoside serves as a precursor of coumarin and acts as an important chemical signaling molecule for leguminous crops to recruit rhizobia (Qiao et al., 2024). Its persistent presence in the crop rhizosphere suggests that the legacy during the green manure growth and decomposition exerts a long-term regulatory effect on the subsequent rice rhizosphere microbiome (Barel et al., 2018). This reveals the cross-seasonal persistence mechanism of green manuring effects and provides chemical evidence for understanding the ecological memory in green manure-rice rotation systems. Furthermore, constructed random forest models using various enriched metabolites subset to identify the key individual metabolites in soil nitrogen component accumulation. Metabolites specifically enriched in the GM2 treatment explained 65.75% of the variation in AHN accumulation, which was substantially higher than that explained by GM1 specific (30.04%), GM3 specific (1.53%), or shared metabolites (26.9%) (**Supplementary Table S3**). In contrast, the accumulation of MIN was driven by a more limited set of metabolites, with the optimal random forest model explaining 42% of its variation. Among these effectors, GlcN-S (an amino sugar-containing metabolite), and GSSG (a small peptide), may act as key signaling molecules that mediate cross-kingdom communication between plants and microorganisms, thereby enhancing the transformation of (Tan et al., 2024). Among the taxa significantly associated with multiple metaMIN, *Desulfuromonadia*, *Desulfobacteria*, and *Thermodesulfovibrionia* are primarily known for their roles in sulfur cycling. Notably, under anaerobic conditions, they also exhibit substantial heterotrophic denitrification potential, thereby influencing soil nitrate nitrogen dynamics (Wang S et al., 2024).

The rhizosphere metabolites associated with AHN accumulation are primarily originated from three distinct sources: xenobiotics, secondary metabolites, and primary metabolites. The xenobiotics including CThzMOI, tBuO-Azp(5-oxo)-COOEt, BMATE, 5,6,7,8-THQ, and Folpet made significant contributions to AHN accumulation. These metabolites are not naturally produced by soil organisms or crops. Instead, they act on soil microorganisms, subsequently influencing soil nutrient transformation and cycling accumulation by either inhibiting or stimulating the microbial communities involved in nutrient cycling. The secondary metabolites, including 10-HDA, Cyclo(Leu-Pro), Piperidine, Sarcosine, and Kahweol function both as chemical signal for the direct mobilization and release of nutrients and as biological regulators that restructure microbial community composition and activity, ultimately enhancing soil carbon and nitrogen cycling efficiency (Singh et al., 2025; Haichar et al., 2014). Primary metabolites such as L-isoleucine and GSSG are essential molecules for microbial growth and are considered to mediate resource exchange that attracts specific functional microbial taxa to the crop rhizosphere. By serving as enzyme constituents, providing energy, and mobilizing nutrients, these metabolites directly drive the cycling of carbon and nitrogen (Kost et al., 2023).

Multiple metabolites associated with AHN accumulation were significantly and positively correlated with bacterial taxa including *Gammaproteobacteria*, *Negativicutes*, and *Syntrophomonadia*, as well as with fungal classes such as *Sordariomycetes* and *Endogonomycetes*. *Gammaproteobacteria* and *Sordariomycetes*, the representative taxa with well-established roles in organic matter decomposition and nitrogen cycling (Fu et al., 2025; Liu et al., 2025), were enriched in the rhizosphere under the regulation of crop root metabolites. This enrichment may mediate the expansion of the soil organic nitrogen pool. As evidence, PLS-PM further confirmed that the metabolite-driven accumulation of AHN was indirectly mediated via the bacterial pathway (standardized effect = 0.838, *P* < 0.05), while the fungal pathway effect was not significant.

Moreover, metabolites also make direct contribution to AHN accumulation. For MIN, accumulation was directly associated with the corresponding metabolites, which also significantly enriched the associated bacterial taxa. However, the pathways through which metabolites drive increased MIN accumulation by microorganisms are not sequentially linked, as bacterial groups do not exhibit significant direct effects driving MIN accumulation. These findings suggested that the pathways linking metabolites, microbes and soil N pools plays a dominant role in shaping the soil labile organic nitrogen pool. And the metabolites contribute simultaneously to organic nitrogen accumulation and inorganic nitrogen transformation by acting as both chemical signals and molecular substrates. Thus, providing explicit functional targets for future targeted regulation of the rhizosphere microbiome to enhance soil nitrogen pool sustainability.

## Conclusion

Green manuring with 20% nitrogen fertilizer reduction achieved stable yield while significantly expanding soil organic nitrogen pools, while green manuring without nitrogen reduction accelerated depletion of soil organic nitrogen. Green manuring enriched nitrogen cycling-related bacteria and saprotrophic fungi, and increased the enzyme activities involved in organic nitrogen depolymerization. Rhizosphere metabolomic profiles reshaping emerged as a key role linking green manuring to microbial recruitment and nitrogen transformation. Enriched metabolites, particularly amino acids and signaling-related compounds, strongly associated with nitrogen-cycling microbial taxa and played important roles in regulating organic and inorganic nitrogen accumulation. Our findings demonstrate that coordinated rhizosphere metabolite–microbiome interactions under green manuring and nitrogen reduction can simultaneously sustain rice production and improve soil nitrogen stability, providing a promising strategy for sustainable paddy management.

## Data Availability

The datasets presented in this study can be found in online repositories. The names of the repository/repositories and accession number(s) can be found in the article/**Supplementary Material**.
