# Supplementary material for: Green manuring combined with nitrogen reduction sustained rice productivity and expanded soil organic nitrogen pool via promoting coordinated rhizosphere metabolite–microbiome interactions
Source: Front Plant Sci. 2026 Sep 30;17:1966893. doi: 10.3389/fpls.2026.1966893 (PMC13662682; doi:10.3389/fpls.2026.1966893)
Supplement: Supplementary file 1 [file DataSheet1.docx]

**Supplementary methods**

**Measurement of the components of soil organic nitrogen**

Briefly, air-dried soil samples were passed through a 2-mm sieve to remove roots and stones; a sub-sample was further ground to pass a 0.15-mm mesh for chemical analysis. For acid hydrolysis, 1.00 g of sieved soil was hydrolyzed with 15 mL of 6 M HCl in an autoclave at 121 °C (15 psi) for 6 h. After cooling, the hydrolysate was transferred to a 50 mL volumetric flask, diluted to volume with deionized water, and clarified by centrifugation or filtration. Acid-hydrolyzable nitrogen (AHN) was determined by micro-Kjeldahl digestion of the hydrolysate. Ammonium nitrogen (AN), amino acid nitrogen (AAN), and amino sugar nitrogen (ASN) were determined by steam distillation after reacting the hydrolysate with MgO, ninhydrin, and phosphate-borate buffer (pH 11.2), respectively. Finally, non-hydrolyzable nitrogen (NHN) was calculated as the difference between TN and AHN, while hydrolyzable unidentified nitrogen (HUN) was determined by subtracting the sum of AN, AAN, and ASN from AHN.

**Measurement of Soil enzyme activities**

For all assays, 0.1 g of fresh soil was weighed into centrifuge tubes. To inhibit microbial proliferation, samples for urease, protease, and LAP assays were pre-treated with toluene (25, 5, and 25 μL, respectively) and equilibrated at room temperature for 15 min. **Urease** activity was determined by adding 125 μL of 10% (w/v) urea solution and 250 μL of citrate buffer (pH 6.7) to the toluene-treated soil, followed by incubation at 37 °C for 24 h. To quantify ammonium release, 72 μL of the supernatant was mixed sequentially with 24 μL of sodium phenate and 24 μL of sodium hypochlorite in a 96-well plate. After color development for 20 min at room temperature, 80 μL of deionized water was added, and the absorbance was measured at 630 nm. **Protease** activity was assayed by incubating the soil with 50 μL of buffer and 100 μL of casein solution at 50 °C for 48 h with periodic agitation. The reaction was terminated with 100 μL of trichloroacetic acid (TCA) solution before centrifugation. Tyrosine release was quantified by mixing 40 μL of the supernatant with 160 μL of Na_2_CO_3_ solution and 40 μL of Folin–Ciocalteu reagent. The mixture was incubated at 40 °C for 10 min, centrifuged again (10000×g, 10 min), and 200 μL of the resulting supernatant was transferred to a 96-well plate to read the absorbance at 680 nm. **NAG** activity was evaluated by mixing the soil with 237.5 μL of citric acid–disodium hydrogen phosphate buffer (pH 5.0) and 62.5 μL of 4-Nitrophenyl 2-acetamido-2-deoxy-β-D-glucopyranoside (PNP-NAG) solution. After incubation at 37 °C for 3 h and subsequent centrifugation at 25 °C, a 70 μL aliquot of the supernatant was mixed with 140 μL of 0.5% (w/v) NaOH to terminate the reaction. The mixture was left at room temperature for 2 min, and 200 μL was transferred to a 96-well plate for absorbance measurement at 400 nm. **LAP** activity was determined by adding 425 μL of Tris-HCl buffer and 50 μL of L-Leucine-p-nitroanilide solution to the soil, followed by incubation at 37 °C for 3 h. For the control groups, the substrate was replaced with an equal volume of acetone. Following centrifugation, 200 μL of the supernatant was transferred to a 96-well plate, and the absorbance was recorded at 405 nm.

**Supplementary figures**

**Fig S1 Profiles of bacterial and fungal composition in the rice soil–root continuum.**

**Fig S2 Profiles of bacterial and fungal α-diversity.**

**Fig S3 Differences in bacteria in bulk soil across different treatments at phylum level**

**Fig S4 Differences in bacteria in bulk soil across different treatments at genus level**

**Fig S5 Differences in bacteria in rhizosphere soil across different treatments at phylum level**

**Fig S6 Differences in bacteria in rhizosphere soil across different treatments at genus level**

**Fig S7** **Differences in bacteria in root endosphere across different treatments at phylum level**

**Fig S8 Differences in bacteria in root endosphere across different treatments at genus level**

**Fig S9 Differences in fungi in bulk soil across different treatments at phylum level**

**Fig S10 Differences in fungi in bulk soil across different treatments at genus level**

**Fig S11 Differences in fungi in rhizosphere soil across different treatments at phylum level**

**Fig S12 Differences in fungi in rhizosphere soil across different treatments at genus level**

**Fig S13 Differences in fungi in root endosphere across different treatments at phylum level**

**Fig S14Differences in fungi in root endosphere across different treatments at genus level**

**Fig S15 Characterization of metabolic pathways in the rice rhizosphere**

**Fig S16 Procrustes analysis evaluating the relationships between rhizosphere metabolite composition and microbial population in bulk soil**

**Fig S17 Procrustes analysis evaluating the relationships between rhizosphere metabolite composition and microbial population in root endosphere**

**Fig S18 Relative abundance and differential analysis of metabolites across treatments**

**Fig S19 Overview of metabolite enrichment under different green manuring treatments compared to fallow**

**Fig S20 Hierarchical classification of rhizosphere metabolites under the F treatment**

**Fig S21 Hierarchical classification of rhizosphere metabolites under the GM1 treatment**

**Fig S22 Hierarchical classification of rhizosphere metabolites under the GM2 treatment**

**Fig S23 Hierarchical classification of rhizosphere metabolites under the GM3 treatment**

**Fig S24 Enrichment of amino acid metabolites in green manure treatments compared with F treatment**

**Fig 25 Enrichment of carbohydrate metabolites in green manure treatments compared with F treatment**

**Fig S26 Enrichment of fatty acids metabolites in green manure treatments compared with F treatment**

**Fig S27 Linear regression analysis between key metabolites and Acid-hydrolyzable nitrogen (AHN)**

**Fig S28 Linear regression analysis between key metabolites and mineral nitrogen (MIN)**
